# Genome-wide transcriptional profiling of *Botrytis cinerea* genes targeting plant cell walls during infections of different hosts

**DOI:** 10.3389/fpls.2014.00435

**Published:** 2014-09-03

**Authors:** Barbara Blanco-Ulate, Abraham Morales-Cruz, Katherine C. H. Amrine, John M. Labavitch, Ann L. T. Powell, Dario Cantu

**Affiliations:** ^1^Department of Viticulture and Enology, University of California, DavisDavis, CA, USA; ^2^Department of Plant Sciences, University of California, DavisDavis, CA, USA

**Keywords:** *Botrytis*, noble rot, plant pathogenic fungi, CAZymes, RNAseq, tomato, grape, lettuce

## Abstract

Cell walls are barriers that impair colonization of host tissues, but also are important reservoirs of energy-rich sugars. Growing hyphae of necrotrophic fungal pathogens, such as *Botrytis cinerea* (*Botrytis*, henceforth), secrete enzymes that disassemble cell wall polysaccharides. In this work we describe the annotation of 275 putative secreted Carbohydrate-Active enZymes (CAZymes) identified in the *Botrytis* B05.10 genome. Using RNAseq we determined which *Botrytis* CAZymes were expressed during infections of lettuce leaves, ripe tomato fruit, and grape berries. On the three hosts, *Botrytis* expressed a common group of 229 potentially secreted CAZymes, including 28 pectin backbone-modifying enzymes, 21 hemicellulose-modifying proteins, 18 enzymes that might target pectin and hemicellulose side-branches, and 16 enzymes predicted to degrade cellulose. The diversity of the *Botrytis* CAZymes may be partly responsible for its wide host range. Thirty-six candidate CAZymes with secretion signals were found exclusively when *Botrytis* interacted with ripe tomato fruit and grape berries. Pectin polysaccharides are notably abundant in grape and tomato cell walls, but lettuce leaf walls have less pectin and are richer in hemicelluloses and cellulose. The results of this study not only suggest that *Botrytis* targets similar wall polysaccharide networks on fruit and leaves, but also that it may selectively attack host wall polysaccharide substrates depending on the host tissue.

## Introduction

The cell wall matrix is one of the first and largest plant structures that pathogens encounter when interacting with potential hosts. The composition and architecture of cell walls vary between plant species, organs and developmental stages. Two co-extensive networks of polysaccharides comprise up to 80% of the mass of most plant cell walls. A network of cellulose microfibrils is cross-linked via hydrogen-bonded hemicelluloses and is embedded within a second network, a matrix of simple and branched pectin polysaccharides (Carpita and Gibeaut, 1993). The integrity of the hemicellulose-cellulose microfibril network provides much of the strength and rigidity of the cell wall (Harris and Stone, 2008; Scheller and Ulvskov, 2010). The pectin network influences the wall's porosity and provides structural coherence (Ishii et al., 2001; Vincken et al., 2003). Homogalacturonan (HG) and rhamnogalacturonans (RG-I and RG-II) are the major pectins of the primary walls of dicots and non-graminaceous monocots (Voragen et al., 2009). Pectins are important for cell-to-cell adhesion and they are particularly abundant in the middle lamella and corners between adjacent cells (Mohnen, 2008). Structural glycoproteins, soluble proteins, ions and metabolites are also located within the polysaccharide networks of most cell walls (Cassab, 1998; Keegstra, 2010).

Plant cell walls that are recalcitrant to decomposition by microorganisms and walls that favor the timely activation and correct allocation of host defenses are more likely to resist pathogen infections (Cantu et al., 2008a; Underwood, 2012). For example, plant cell wall-associated kinases and receptors are crucial to sense invading pathogens and to promptly induce immune responses, including structural reinforcements of the wall and production of anti-pathogen compounds (Cantu et al., 2008a; Hematy et al., 2009). Furthermore, pre-formed defense proteins (e.g., extracellular pathogenesis-related proteins) and their locations within the plant cell wall matrix contribute to processes that prime host tissues for resistance (Powell et al., 2000; Cantu et al., 2008b).

Necrotrophic pathogens, such as *Botrytis*, have evolved complex strategies to overcome the plant immune system (Weiberg et al., 2013) and to destroy the pectin-rich middle lamellae and primary cell walls of the host, inducing cell death and compromising the integrity of host tissues (Tiedemann, 1997; Van Baarlen et al., 2004; Cantu et al., 2008a; Curvers et al., 2010). *Botrytis* is considered a generalist pathogen because it is capable of infecting a wide variety of plant hosts and organs. During infections, *Botrytis* secretes diverse proteins and enzymes that modify the host cell walls (Van Kan, 2006; Zhang and van Kan, 2013a). Some of these proteins, such as the polygalacturonase BcPG1, have been demonstrated to be important virulence factors in multiple host tissues (Ten Have et al., 1998; Valette-Collet et al., 2003; Espino et al., 2005; Kars et al., 2005a; Brito et al., 2006; Nafisi et al., 2014). Characterizing the cell wall-degrading enzymes deployed by *Botrytis* on different hosts and tissues may help identify virulence functions that *Botrytis* uses on all hosts and those that are important on specific hosts, organs, or developmental stages.

The Carbohydrate-Active enZymes (CAZymes) are proteins with predicted catalytic and carbohydrate-binding modules that degrade, modify, or create glycosidic bonds. Therefore, some CAZymes are candidates for proteins that participate in the modification and breakdown of cell wall polysaccharides (Cantarel et al., 2009). The assignment of a gene to a particular CAZyme family can predict the catalytic properties of the protein it encodes and its possible substrates (Cantarel et al., 2009; Park et al., 2010). Sequence homology to known CAZyme genes in combination with computational prediction of protein secretion (SignalP; Petersen et al., 2011) has been used extensively for *in silico* identification and classification of the repertoire of cell wall degrading enzymes of pathogenic fungi with sequenced genomes (Floudas et al., 2012; Suzuki et al., 2012; Blanco-Ulate et al., 2013a,b,c,d).

Genome-wide transcriptional profiling approaches have been applied successfully to study the regulation of pathogen virulence factors in plant hosts (Noël et al., 2001; Ithal et al., 2007; Jeon et al., 2007; O'Connell et al., 2012; Schmidtke et al., 2012; Cantu et al., 2013; Wiemann et al., 2013; Zhang et al., 2013). In this study we (i) identified in the current release of the publically available *Botrytis* genome (strain B05.10 v.1; Amselem et al., 2011) genes encoding putatively secreted CAZymes, (ii) analyzed the phylogenetic relationships of these genes, and (iii) profiled their expression when *Botrytis* interacts with three plant hosts. The plant hosts chosen for this study, ripe tomato fruit, ripe grape berries and lettuce leaves, represent to important post-harvest commodities, which are highly susceptible to infections by *Botrytis*. Our results suggest that *Botrytis* not only expresses a rich repertoire of activities that target the many diverse structures of the plant cell walls, but also that some of these functions are differentially regulated depending on the host.

## Materials and methods

### Annotation of *Botrytis* CAZymes

Transcriptome sequences of *Botrytis cinerea* (strain B05.10 v.1; Amselem et al., 2011) were obtained from http://www.broadinstitute.org/annotation/genome/botrytis_cinerea. The transcriptome was annotated for sequences encoding Carbohydrate-Active enZymes (CAZymes; http://www.cazy.org) with the CAZymes Analysis Toolkit (http://mothra.ornl.gov/cgi-bin/cat/cat.cgi; Park et al., 2010) with an e value < 1e-2, a bit score threshold of 55 and a rule level of support of 40. Functional annotation of the CAZymes genes was carried out with Blast2GO v.2.7.1 (http://www.blast2go.com/start-blast2go; Götz et al., 2008), which performed a BLASTx search against the non-redundant (nr) protein database of NCBI; default parameters were used. The predicted CAZymes from *Botrytis* were then clustered in protein tribes based on sequence similarities using BLASTp alignments (*e*-value <1e−6) and Tribe-MCL (Enright et al., 2002) following methods described in Haas et al. (2009). The presence of secretion signal peptides was evaluated for all genes in the transcriptome using SignalP v.4.0 (http://www.cbs.dtu.dk/services/SignalP-4.0/; Petersen et al., 2011) with the following parameters: 0.50 D-cutoff values for SignalP-TM and 0.45 for SignalP-noTM.

One of the limitations of *in silico* analyses of secretion peptides is the occurrence of false positives and false negatives (Petersen et al., 2011; Melhem et al., 2013). SignalP v.4.0 was reported to have a higher false negative rate (8.80%) than false positive rate (3.30%) when predicting secretion signals in plant proteins (Melhem et al., 2013). A literature search of previously validated secreted *Botrytis* CAZymes was performed to identify possible false negatives resulting from the SignalP prediction. Two CAZyme-encoding genes, *BcPME1* (*BC1G_06840*) and *BcXyn11A* (*BC1G_02167*), had SignalP prediction scores below the 0.50/0.45 D-cutoff values, but both genes have been experimentally proven to encode secreted proteins (Valette-Collet et al., 2003; Kars et al., 2005b; Brito et al., 2006; Shah et al., 2009a,b; Fernández-Acero et al., 2010; Li et al., 2012); hence, they were included in the dataset of secreted *Botrytis* CAZymes. The existence of other false negatives still needs to be experimentally evaluated for each CAZyme that did not pass the SignalP thresholds.

### Phylogenetic analyses

The protein sequences of 7 CAZyme subfamilies including genes with putative roles in degrading plant cell walls, based on manual curation of CAZymes and functional annotations, were analyzed. Multiple global sequence alignments were conducted with MUSCLE (Edgar, 2004) for all protein sequences in a particular tribe using default parameters. Phylogenetic analyses were conducted in MEGA v.5.2.2 (Tamura et al., 2011) using the Neighbor-Joining method with 1000 bootstrap replicates (Felsenstein, 1985; Saitou and Nei, 1987). All positions containing gaps and missing data were eliminated.

### Biological material

Tomato (*Solanum lycopersicum*) cv. Ailsa Craig was provided by the Tomato Genetics Research Center (UC Davis). Tomato plants were grown in the field in Davis, California. Fruit were tagged at 3 days post-anthesis (dpa) and harvested at the red ripe stage (42 dpa). The ripening stages of the fruit were confirmed by color, size and texture measurements.

The *Botrytis* strain B05.10 used to inoculate the tomato fruit was provided by Dr. J. A. L. van Kan (Department of Phytopathology, Wageningen University). Conidia were collected from sporulating cultures grown on 1% potato dextrose agar. Tomato fruit were disinfected and inoculated as in Cantu et al. (2008a). At the time of inoculation, fruit were wounded at seven sites to a depth of 2 mm and a diameter of 1 mm. Wounded sites were inoculated with 10 μL of a water suspension containing 5000 conidia. All fruit samples were incubated at 20°C in high humidity for 3 days. When material was collected for analysis, the tomato fruit were deseeded, frozen, and ground to fine powder in liquid nitrogen. Three biological replicates were produced; each replicate was an independent pool of 8–10 *Botrytis*-infected tomato fruit.

Ripe (23 brix) grape berries (*Vitis vinifera* cv. Sémillon) showing the initial symptoms of *Botrytis* infections were collected from the Dolce Winery Vineyards (Napa Valley, California). Fruit were field inoculated by spraying conidia of the *Botrytis* strain BcDW1 (Blanco-Ulate et al., 2013a). Transcript polymorphisms detected in the RNAseq data suggest that other strains also infected the berries in the vineyard (results not shown). Determination of the initial stage of *Botrytis* infection (S1) was based on the time at which individual berries showed a partial color change from green to pink, but the berries still maintained their turgor and tissue integrity. The S1 stage of botrytized-grape berries was confirmed by the amount of fungal biomass present in the berries (described below). On the same day of harvest, individual infected grape berries were deseeded, frozen, and ground to fine powder in liquid nitrogen. Four biological replicates were generated from independent pools of 10–15 *Botrytis*-infected grape berries.

### Botrytis biomass determination

Fungal biomass was quantified using the QuickStix Kit for *Botrytis* (EnviroLogix), which utilizes the monoclonal antibody BC12.CA4 (Meyer and Dewey, 2000) as described by Cantu et al. (2008b). One gram of ground tissue (pericarp and epidermis) from each biological replication was suspended in the kit buffer, 1:40 m/v for tomato fruit and 1:20 m/v for grape berries. The amount of material that cross-reacted with the antibody was measured in 500 μL of the tissue suspension. The intensity of the test line was determined with the QuickStix reader (Envirologix) and converted into fungal biomass (μg/gFW of fruit) based on standard curves using known amounts of dry mycelium diluted into extracts of healthy tomato fruit tissue (Cantu et al., 2009a).

### RNA sequencing and data processing

Total RNA was extracted from two grams of infected tissues (pericarp and epidermis) from each biological replicate as described in Blanco-Ulate et al. (2013e). RNA concentration and purity were measured using the NanoDrop 2000c Spectrophotometer (Thermo Scientific). RNA integrity was checked by agarose gel electrophoresis. The Illumina TruSeq RNA Sample preparation Kit v.2 was used to prepare cDNA libraries from 4 μg of total RNA. Libraries were barcoded individually and analyzed with the High Sensitivity DNA Analysis Kit using an Agilent 2100 Bioanalyzer (Agilent Technologies). Sequencing was carried out on an Illumina HiSeq machine at the DNA Technologies Service Core at UC Davis. The Illumina raw reads were deposited in the National Center for Biotechnology Information's Gene Expression Omnibus (GEO) and are accessible through GEO (GSE57588 accession; http://www.ncbi.nlm.nih.gov/geo/query/acc.cgi?acc=GSE57588). Quality trimming of raw reads was done with Sickle v.1.21 (https://github.com/ucdavis-bioinformatics/sickle) with a threshold of 20 (Q >20) and adapter trimming was done with Scythe v.0.991 (https://github.com/ucdavis-bioinformatics/scythe) with a prior of 0.4.

The sequences of the *Botrytis* (strain B05.10 v.1) and grape (v. 12X, http://www.genoscope.cns.fr/externe/Download/Projets/Projet_ML/data/12X/annotation/) transcriptomes were combined and used as a reference for mapping *Botrytis*-infected grape reads. Likewise transcriptomes of *Botrytis* and tomato (ITAG2.3, ftp://ftp.solgenomics.net/tomato_genome/annotation/ITAG2.3_release/) were merged and used as reference for *Botrytis*-infected tomato reads. Bowtie2 v.2.1.0 (Langmead and Salzberg, 2012) was used to align the processed reads to the combined references with the parameters—end-to-end—sensitive. Read counts were extracted from the bowtie2 alignments using the script sam2counts.py v.0.91 (https://github.com/vsbuffalo/sam2counts).

Raw counts of *Botrytis* genes expressed at 2 days post-infection of lettuce leaves were obtained from De Cremer et al. (2013). In this study, lettuce leaves (*Lactuca sativa* cv. Salinas) of 5 week-old plants were inoculated in the growth chamber with a spore suspension of *Botrytis*, strain B05.10 (De Cremer et al., 2013).

### Differential expression analyses

The Bioconductor package DESeq v. 1.14.0 (Anders and Huber, 2010) was used to normalize the raw transcript counts of *Botrytis* genes encoding potentially secreted CAZymes from infected lettuce leaves, ripe tomato fruit and grape berries. The DESeq pipeline was used to (i) compare the expression profiles of potentially secreted CAZymes during *Botrytis* infections of lettuce leaves, ripe tomato fruit and grape berries, and (ii) identify differentially expressed (DE) genes (*P*-adjusted value ≤ 0.05).

### Quantitative reverse transcription PCR (qRT-PCR)

cDNA was prepared from the isolated RNA using the M-MLV Reverse Transcriptase (Promega). qRT-PCR was performed on a StepOnePlus PCR System using Fast SYBR Green Master Mix (Applied Biosystems). All qRT-PCR reactions were performed as follows: 95°C for 10 min, followed by 40 cycles of 95°C for 3 s and 60°C for 30 s. The *BOTRYTIS RIBOSOMAL PROTEIN-LIKE5* (*BcRPL5*, *BC1G_13576*) was used as the reference gene and processed in parallel with the genes of interest. The primer sequences to amplify the *BcRPL5*, *BcPG1*, and *BcPG2* genes were obtained from Zhang and van Kan (2013b). Transcript levels for all genes in this study were linearized using the formula 2^(*BcRPL5* CT−*TARGET* CT)^ as described in Chen and Dubcovsky (2012). Data presented are means of 3–4 biological replicates of infected tomato and grape berries.

## Results

### Predicted CAZyme *Botrytis* genes

The genome of *Botrytis cinerea* (strain B05.10; Amselem et al., 2011) is predicted to encode 1155 CAZymes based on a similarity search against the entire non-redundant sequences of the CAZy database using the CAZYmes Analysis Toolkit (Park et al., 2010; Supplemental Table S1). Putative secretion signals were found in 275 CAZyme *Botrytis* genes (SignalP v4.0; Petersen et al., 2011). Glycoside hydrolases (GHs) were the most abundant class of putative secreted CAZymes (48.72%); among the GHs, the GH28 subfamily was the largest group (14.18% of all GHs). Twenty-three percent of the CAZyme genes encoded carbohydrate-binding proteins (CBMs), 16.48% coded for carbohydrate esterases (CEs) and 8.06 and 3.30% encoded glycosyltransferases (GTs) and polysaccharide lyases (PLs), respectively. Eighty-eight of these CAZy proteins were previously detected in the secretomes of *Botrytis-infected* tomato fruit and *Botrytis* grown in host-free culture systems (Supplemental Table S2; Shah et al., 2009a,b; Espino et al., 2010; Fernández-Acero et al., 2010; Li et al., 2012; Shah et al., 2012.)

Of the 275 potentially secreted CAZymes, 121 were grouped into 39 protein tribes (containing at least two proteins; Supplemental Table S2). Protein tribes are protein families defined by their sequence similarity using the Tribe-MCL algorithm (Enright et al., 2002). Members within a protein tribe are predicted to have similar molecular structures and, thus, may perform similar biochemical functions. In most cases, proteins of the same tribe share a common evolutionary history (Enright et al., 2003). The largest tribes had 6–7 proteins and belonged to the CAZyme subfamilies CE10 (tribes 0 and 2), GH28 (tribe 1), CBM1 (tribe 3), GH3 (tribe 4), and GH16 (tribe 5). Tribe 1 contained the well-characterized endo-polygalacturonases (endo-PGs, BcPG1-6), which cleave the homogalacturonan (HG pectin backbones; Jayani et al., 2005). Tribes 4 and 6 included, respectively, all putative β-glucosidases and xyloglucan (XyG) transglucosylase/hydrolases (XTHs) present in the *Botrytis* genome. Both the β-glucosidases and XTHs classes target the backbones of XyGs; β-glucosidases may be also involved in cellulose degradation (Eklöf and Brumer, 2010).

### RNA sequencing (RNAseq) of *Botrytis*-infected plant tissues

RNAseq was performed to characterize the expression profiles of *Botrytis* genes encoding CAZymes during infections of ripe tomato fruit and grape berries. mRNA was isolated from *Botrytis*-infected tomato fruit (at 3 days post-inoculation, dpi) and grape berries (stage S1; see Methods) and analyzed by high-throughput sequencing. RNAseq data from lettuce leaves 2 days after *Botrytis* infection (De Cremer et al., 2013) were analyzed in parallel to the fruit data, in order to detect commonalities and differences of CAZyme expression between fungal infections of different host organs and species. A summary of parsed reads from each biological replication of the three *Botrytis*-infected plant hosts and the number of reads mapped to the *Botrytis* (strain B05.10) and plant transcriptomes is provided in Table 1.

**Table 1:** **Summary of trimmed and mapped reads of mRNA from *Botrytis*-infected tissues**.

**Samples**	**Quality-trimmed reads (Q >20)**	**Total reads mapped**		**Number of reads uniquely mapped**	
		**Number**	**Percentage (%)**	***Botrytis***	**Plant host**
Lettuce #1	20,726,205	15,476,051	74.67	981,495	14,494,556
Lettuce #2	8,742,262	6,098,894	69.76	324,147	5,774,747
Lettuce #3	7,024,911	5,197,645	73.99	566,381	4,631,264
Tomato #1	15,300,253	12,693,894	82.97	971,765	11,722,129
Tomato #2	15,315,439	12,085,072	78.91	2,428,994	9,656,078
Tomato #3	17,334,445	14,020,196	80.88	2,480,527	11,539,669
Grape #1	21,529,329	16,579,455	77.01	4,083,310	12,496,145
Grape #2	25,706,831	21,673,021	84.31	983,846	20,689,175
Grape #3	22,110,864	18,024,359	81.52	2,490,373	15,533,986
Grape #4	17,372,190	13,796,319	79.42	2,448,717	11,347,602

Transcript reads from infected lettuce leaves were from De Cremer et al. (2013).

**Figure 1:**
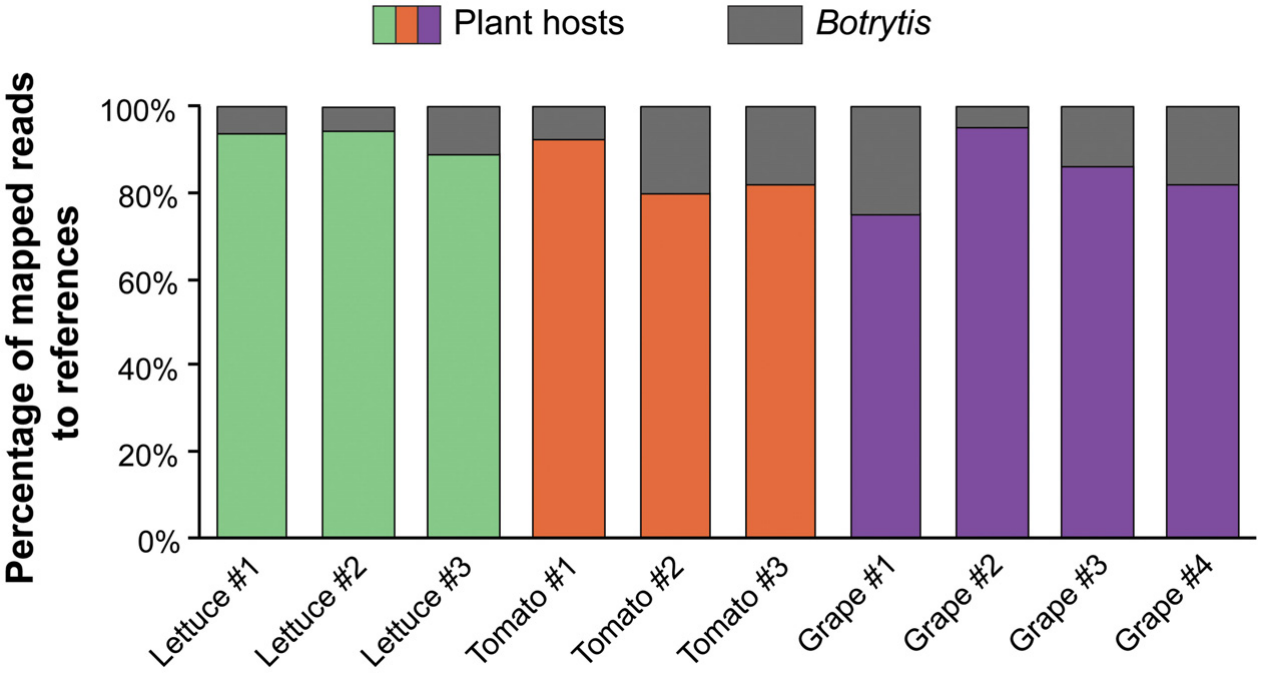
***Botrytis* transcript abundance in infected plant tissues**. Relative composition of RNAseq transcript reads between *Botrytis* and the three plant hosts (i.e., lettuce leaves, tomato fruit and grape berries).

The reads that uniquely mapped to the *Botrytis* transcriptome corresponded to more than 75% of the total predicted genes in the *Botrytis* genome. 12,766 (77.79%), 12,998 (79.21%) and 13,898 (84.69%) *Botrytis* genes were detected in lettuce leaves, tomato fruit, and grape berries, respectively. In addition, the percentage of reads uniquely mapped to the *Botrytis* transcriptome relative to the total number of mapped reads suggested that the amounts of *Botrytis* were comparable between the infected hosts (Figure 1). *Botrytis* infections of ripe tomato fruit at 3 dpi, ripe grapes berries in stage 1 of the noble rot and lettuce leaves at 2 dpi showed similar disease symptoms, which include water-soaked lesions with no or limited fungal sporulation, and without extensive tissue maceration, suggesting that *Botrytis* was at similar stages in its life cycle on the three hosts.

The amounts of *Botrytis* biomass in the infected tomato and grape tissue samples were measured with a monoclonal antibody-based assay (Meyer and Dewey, 2000) and strongly correlated with the number of *Botrytis* transcript reads in the corresponding samples (Supplemental Figure S1). This confirmed that the number of *Botrytis* transcripts from infected plant tissues determined by RNAseq can be used as an indicator of the amount of *Botrytis* biomass present in the infected tissues.

### CAZyme *Botrytis* genes expressed during host plant infections

**Figure 2:**
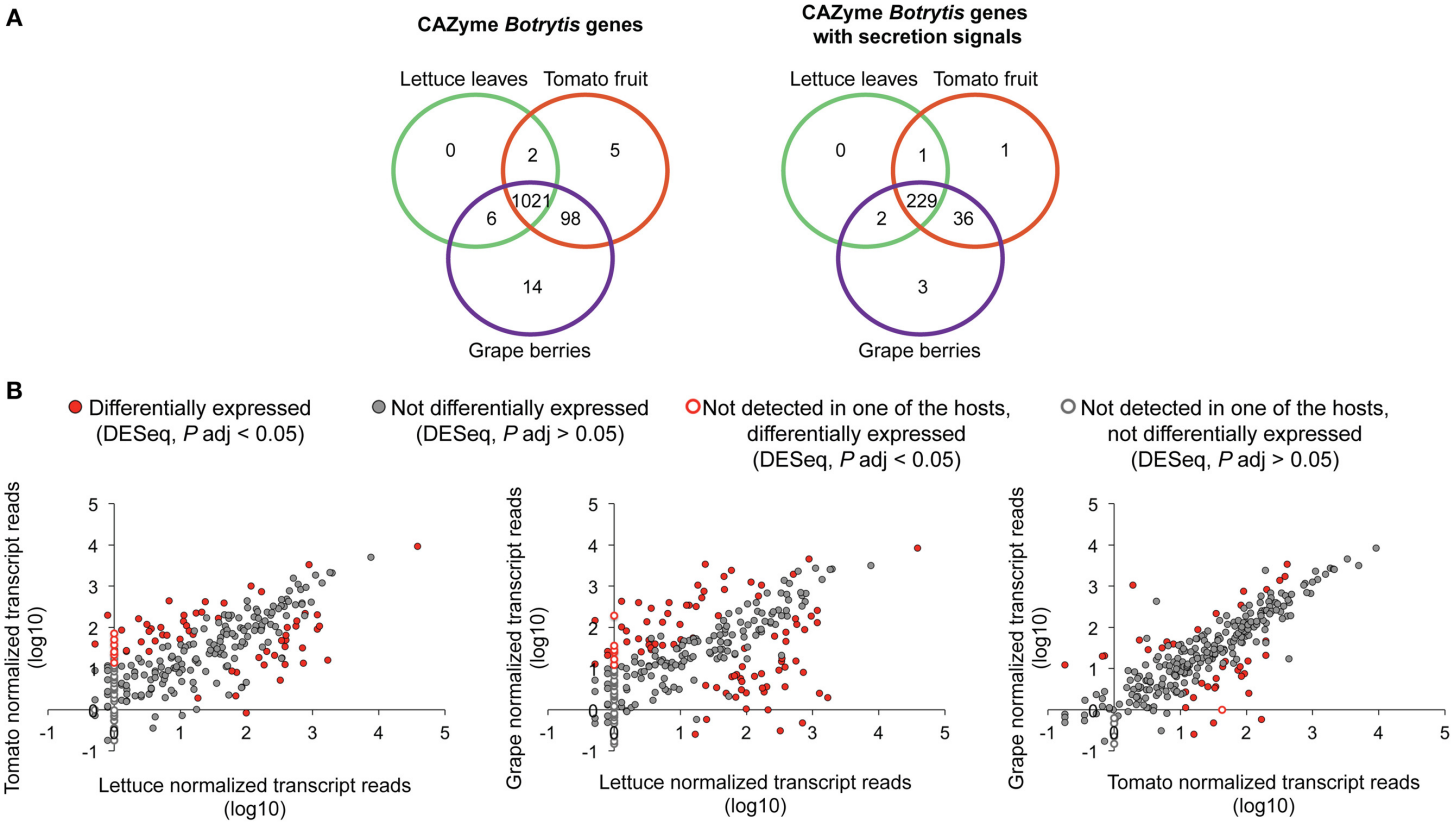
**Comparisons of CAZyme gene expression during infections of lettuce leaves, tomato fruit and grape berries. (A)** Venn diagrams showing the overlapping and unique sets of CAZyme *Botrytis* genes (left) and CAZyme *Botrytis* genes with potential signal peptides (right) detected in the transcriptomes of the three infected host tissues. **(B)** Scatterplots showing all possible pair-wise correlations between DESeq-normalized counts of CAZyme *Botrytis* genes in lettuce, tomato, and grape.

Most (88.40%) of the CAZyme genes predicted in the *Botrytis* genome were detected in the three infected hosts (Figure 2A; Supplemental Table S1). The genes commonly expressed in the three hosts include 83.23% of the CAZyme genes with predicted secretion signals (Figure 2A; Supplemental Table S2). The largest number of genes encoding putative secreted CAZymes belonged to the GH28 and CE10 subfamilies, each with 17 genes (Supplemental Table S2). Although CAZymes secreted by *Botrytis* are expected to target plant cell wall substrates, they could also be involved in remodeling the fungal cell wall as the pathogen grows and develops (Cantu et al., 2009b). Alternatively, they may degrade host cellular contents including starch and glycosylated proteins and secondary metabolites with sugar groups (Faure, 2002; Shah et al., 2009b; Klis et al., 2010). Some CAZymes can act on more than one polysaccharide substrate (Eklöf and Brumer, 2010). Table 2 provides an overview of *Botrytis* CAZymes that might be relevant for the degradation of plant cell walls, as determined by manual curation of their functional annotations.

**Table 2:** **Annotated secreted *Botrytis* enzymes involved in plant cell wall disassembly**.

**Plant cell wall target**	**CAZyme subfamily**	**Functional annotation**	**Proteins potentially secreted**	**Genes expressed in all three hosts**
HG backbone	GH28	Polygalacturonases	11	*BcPG1, BcPG3, BcPG4, BcPG5, BcPG6, BC1G_00240, BC1G_01617, BC1G_11909, BC1G_15118*
	PL1	Pectin lyases	4	*BC1G_07527, BC1G_11690, BC1G_12017, BC1G_12517*
	PL1, PL3	Pectate lyases	4	*BC1G_00912, BC1G_09000, BC1G_07052, BC1G_10229*
	CE8	Pectin methylesterases	3	*BcPME1, BcPME2, BC1G_11144*
RG-I backbone	GH28, GH105|GH28	RG hydrolases	6	*BC1G_01234, BC1G_01923, BC1G_03619, BC1G_03464, BC1G_05961, BC1G_13970*
	GH78	α-L-Rhamnosidases	2	*BC1G_06328*
	CE12	RG acetylesterases	1	*BC1G_14009*
XyG backbone	GH3	β-Glucosidases	6	*BC1G_03179, BC1G_07110, BC1G_07622, BC1G_10231, BC1G_11439, BC1G_14169*
	GH12	XyG-specific β-glucanases	1	*BC1G_00594*
	GH16, GH16|CBM18	Glucanases and XyG transglucosylase/hydrolases	11	*BC1G_00409, BC1G_02932, BC1G_04948, BC1G_07945, BC1G_08924, BC1G_09991*
Xylan backbone	GH10, GH11, GH10|CBM1, GH11|CBM1	β-Xylanases	5	*BcXyn11A, BC1G_00576, BC1G_01778, BC1G_03590*
	GH43	β-Xylosidases	3	*BC1G_02487, BC1G_10797*
Mannans	GH5|CBM1	β-Mannosidases	1	*BC1G_02036*
	GH26, CBM3|GH26| CBM35|GH44	β-Mannanases	2	*BC1G_10341*
Cellulose	GH5, GH5|CBM1, GH45	1,4-β-Glucanases	10	*BC1G_02740, BC1G_03038, BC1G_07822, BC1G_08990, BC1G_09210, BC1G_13855, BC1G_13862, BC1G_16238, BcCEL5A,*
	GH6|CBM1, GH6|CBM2, GH7, GH7|CBM1	Cellulose 1,4-β-cellobiosidases	5	*BC1G_06035, BC1G_08989, BC1G_10880, BC1G_13445, BC1G_14702*
Side-chains/adducts	GH2, GH35	β-Galactosidases	3	*BC1G_02410, BC1G_03567, BC1G_12184*
	GH31	α-Xylosidases	2	*BC1G_12859, BC1G_11115*
	GH43, GH93	α-L-1,5-Arabinanases	2	*BcAra1, BC1G_13938*
	GH47, GH92	α-Mannosidases	4	*BC1G_00245, BC1G_00455, BC1G_09742, BC1G_12174*
	GH51, GH54|CBM42, GH62|CBM13	α-Arabinofuranosidases	4	*BC1G_04994, BC1G_08372, BC1G_10789, BC1G_12138*
	GH53	AG β-galactosidases	1	*BC1G_16209*
	GH95	α-L-Fucosidases	1	*BC1G_08975*
	GH115	Xylan α-1,2-glucuronosidases	1	*BC1G_13153*

The secreted CAZyme-coding genes that were commonly expressed during Botrytis infections of lettuce leaves, tomato fruit and grape berries are presented.

Remarkably, 98.90% of the CAZyme *Botrytis* genes with secretion signals were detected when the data of the transcriptomes from all the hosts were combined (Supplemental Table S2). Only three genes, *BC1G_09963* (CBM50|CBM18|GH18 subfamily), *BC1G_13488* (putative XTH, GH16 subfamily; tribe 5) and *BC1G_13714* (CE10 subfamily; tribe 0) were not found in any of the *Botrytis*-infected tissues.

CAZyme *Botrytis* genes that were detected on some but not all hosts (Supplemental Table S2) were identified. Thirteen percent of the genes encoding putative secreted CAZymes were expressed in infected ripe tomato fruit and grape berries but were not detected in infected lettuce leaves (Figure 2A; Supplemental Table S2). For example, three members of the GH16 subfamily (including two putative XTHs from tribe 5) were detected only on tomato fruit and grape berries. The well-described PG-coding gene *BcPG2* (Kars et al., 2005a) was detected only in infected lettuce leaves and tomato fruit. Two genes (*BC1G_05377* from the CBM18 subfamily and *BC1G_15017* from the AA9 subfamily) were identified only in the transcriptomes of infected lettuce leaves and grape berries.

Besides the *Botrytis* genes that appeared to be preferentially expressed in infected fruit but not in infected lettuce leaves, a few other genes seemed to be specifically expressed in a particular plant species (Supplemental Table S2). Three genes were uniquely detected in infected ripe grape berries: a putative XTH (*BC1G_09829*, GH16 subfamily and tribe 5), a candidate copper-dependent lytic polysaccharide monooxygenase (LPMO, *BC1G_00922*, AA9 subfamily) and a predicted FAD-binding oxidoreductase (*BC1G_06334*, GT22 subfamily). One gene, a putative endo-β-1,4-xylanase (*BC1G_13645*, GH11 subfamily), was detected only in tomato fruit. No CAZyme *Botrytis* genes were detected exclusively in lettuce leaves.

The lowest number of CAZyme genes was detected in *Botrytis*-infected lettuce leaves (Figure 2A, Supplemental Tables S1 and S2). This observation could indicate that *Botrytis* expressed a smaller set of genes in lettuce leaves, or that differences in the experimental design (e.g., inoculations in laboratory conditions vs. field infections) or sequence coverage affected the detection levels. Some (23 genes) of the *Botrytis* genes that were not detected in infected lettuce leaves were genes with low levels (<0.01%) of mapped reads in the other plant hosts, suggesting that these genes may not have been detected in lettuce leaves perhaps because of low coverage (Figure 2B). However, other *Botrytis* genes missing from the infected lettuce transcriptome (4 genes) had moderate to high levels of expression (0.05–0.30%) in fruit tissues, indicating that they may not be relevant during lettuce infections (Figure 2B).

### Relative gene expression of *Botrytis* CAZymes with signal peptides in different host tissues

**Figure 3:**
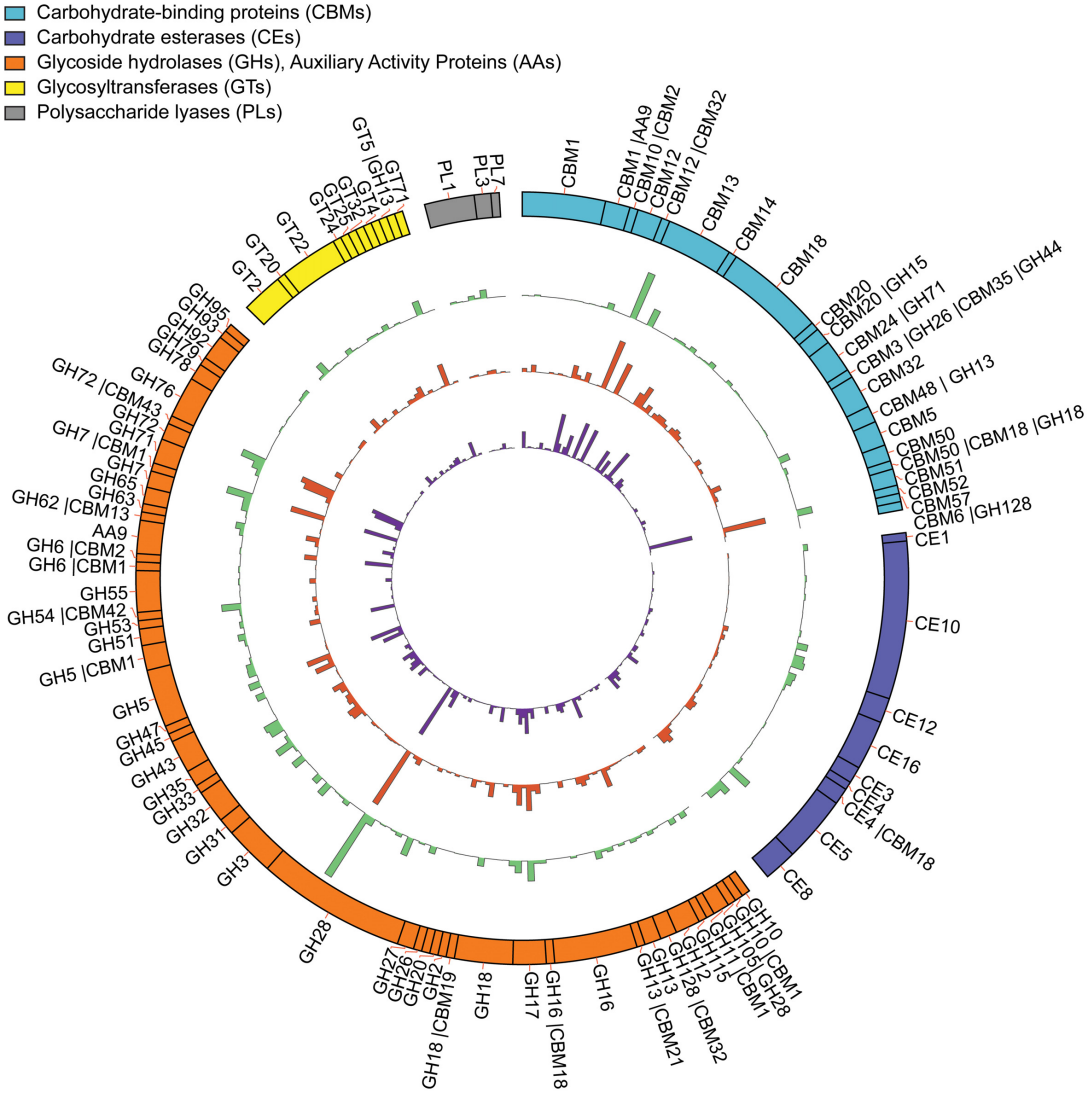
***Botrytis* genes encoding predicted secreted CAZymes and their relative expression levels during infections of three plant hosts**. The outermost ring represents all of the secreted CAZymes predicted in the *Botrytis cinerea* genome (strain B05.10). The inner four rings represent the relative expression of each CAZyme gene during *Botrytis* infections of lettuce leaves (green, second ring from outside), ripe tomato fruit (red, third ring from outside) and ripe grape berries (purple, the innermost ring). The relative expression of each gene is the log2-transformed percentage of normalized reads among the total normalized reads from all CAZyme *Botrytis* genes that possess a secretion signal peptide.

Figure 3 describes the repertoire of potentially secreted CAZymes encoded in the *Botrytis* genome and their relative levels of expression (i.e., percentage of DESeq-normalized reads) when compared to the normalized expression of all CAZyme *Botrytis* genes with signal peptides in a given infected host tissue. Among the most highly expressed *Botrytis* genes coding for characterized or candidate plant cell wall modifying enzymes in each of the plant hosts, *BcPG1* gene had the maximum level of expression during *Botrytis* infections on all hosts (46% of reads in infected lettuce, 18.77% of reads in infected tomato fruit and 13.16% in botrytized-grape berries; Supplemental Table S2). Five other *Botrytis* genes also were highly expressed in the three hosts: a putative cellobiohydrolase gene (*BC1G_14702*, GH7|CBM1 subfamily), a candidate α-xylosidase gene (*BC1G_12859*, GH31 subfamily), *BcPME2* coding for a pectin methylesterase (PME, CE8 subfamily; Kars et al., 2005b), *BcCel5A* encoding an endo-β-glucanase (GH5 subfamily, Espino et al., 2005), and a putative endo-glucanase gene (*BC1G_13862*, GH45 subfamily).

Elevated expression (>0.50% of reads) of six genes encoding CAZymes with defined or putative roles in cell wall degradation was detected during infections of ripe fruit (tomato and grape), but not during infections of lettuce leaves (Supplemental Table S2). These putative fruit-specific genes include two genes encoding putative copper-dependent LPMOs (*BC1G_07653* and *BC1G_07658*, AA9 subfamily, *P*-adjusted value <0.005).

Eight genes were highly expressed in lettuce leaves, but were expressed at lower levels in ripe fruit (Figure 3, Supplemental Table S2). These included a candidate α-L-arabinofuranosidase (*BC1G_04994*, GH54|CBM42 subfamily, *P*-adjusted value <0.001), a predicted exo-PG (*BC1G_01617*, GH28 subfamily, *P*-adjusted value <0.001), and a possible β-glucosidase (*BC1G_07110*, GH3 subfamily, *P*-adjusted value <0.001).

### Association between evolutionary history and expression levels of *Botrytis* proteins involved in plant cell disassembly

**Figure 4:**
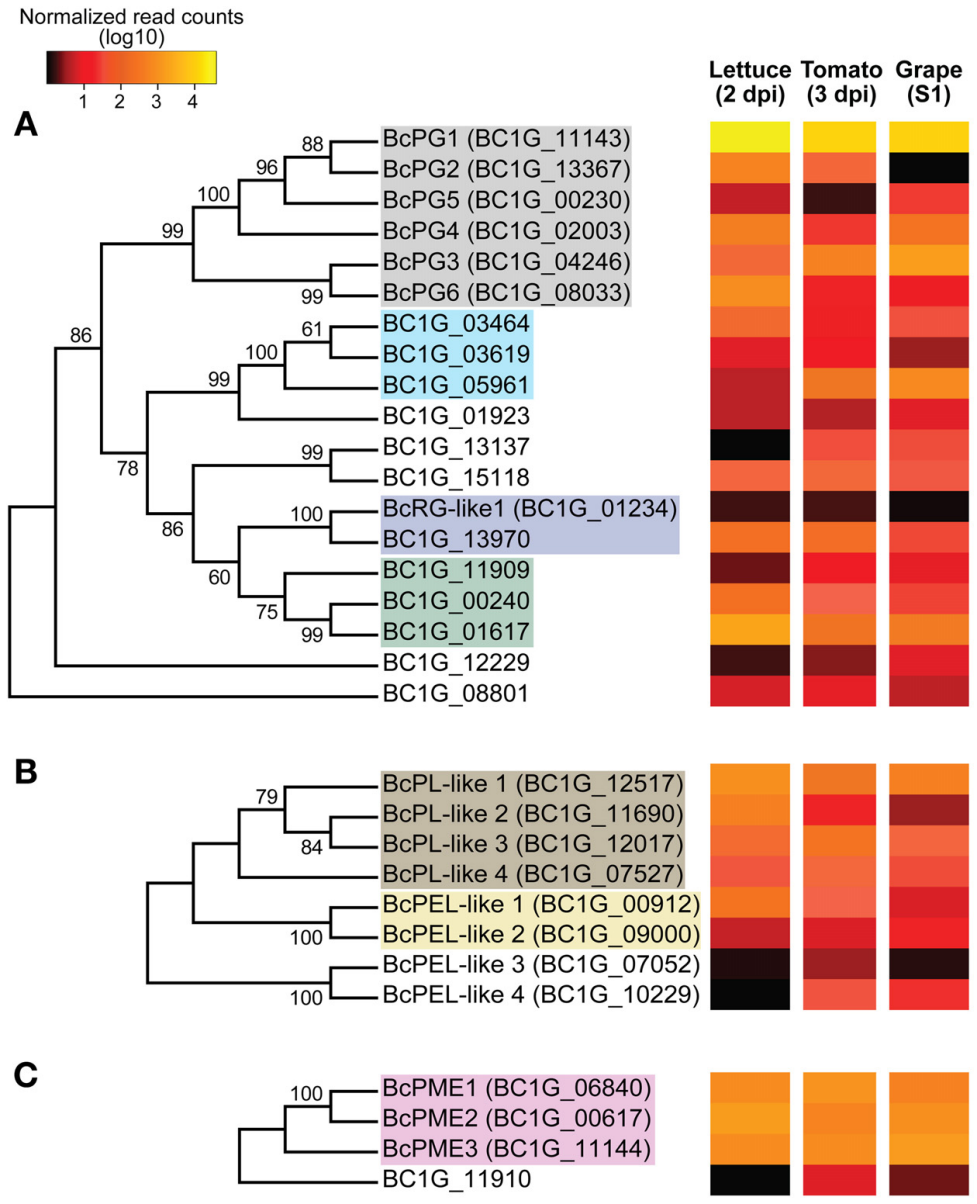
**Phylogenetic tree and expression levels of characterized and candidate enzymes that act on pectin backbones**. Bootstrap consensus trees inferred from 1000 replicates using the Neighbor-Joining method for the *Botrytis* CAZymes with predicted signal peptides in the GH28 **(A)**, PL1 and PL3 **(B)** and CE8 **(C)** subfamilies. The percentages (>50%) of replicated trees in which the associated taxa clustered together in the bootstrap test are shown next to the branches. Colored-boxes in the phylogenetic trees indicate protein tribes (>2 members) determined by BLASTp alignments (*e*-value < 1e−6) and Tribe-MCL (Enright et al., 2002). Boxes in **(A)**, gray = tribe 1, light blue = tribe 16, purple = tribe 26 and green = tribe 10. Boxes in **(B)** brown = tribe 8, yellow = tribe 24. In **(C)**, pink box = tribe 11 (Supplemental Table S2). The colors in the heat maps represent the numbers of DESeq-normalized transcript counts (log10) of the *Botrytis* genes in infected lettuce leaves, ripe tomato fruit and grape berries.

The evolutionary relationships among members of seven CAZyme subfamilies corresponding to characterized and putative cell wall modifying enzymes were inferred using the Neighbor-Joining method (Saitou and Nei, 1987; Figures 4, 6, 7; see Methods for details). The CAZyme subfamilies were chosen based on their functional annotations with the additional requirement of including protein tribes with more than three members.

The phylogenetic analyses of the GH28 subfamily, which included *Botrytis* proteins involved in the hydrolysis of pectins; i.e., HG and RG-I backbones, identified two main clades (86% bootstrap value; Figure 4A). The first clade included the endo-PGs (tribe 1). The second clade was composed of two well-supported groups (79% bootstrap value); one group included the rhamnogalacturonan hydrolases (RGases) of the tribe 16, and the other group was comprised of exo-PGs and RGases from tribes 10 and 26, respectively. The consensus phylogenetic tree of the pectin lyases (PLs) and pectate lyases (PELs) from in the PL1 and PL3 subfamilies (Figure 4B) did not identify distinct clades, but confirmed the classification between protein tribes. For example, the PLs belonging to the tribe 8 group and the PELs, some of which corresponded to tribe 24, were separated in the tree. The PMEs from the CE8 subfamily (Figure 4C) showed a well-supported cluster (100% bootstrap value) composed of the characterized BcPME1 and BcPME2 proteins; however, the tree also supported the grouping of the tribe 11, which also included BcPME3.

**Figure 5:**
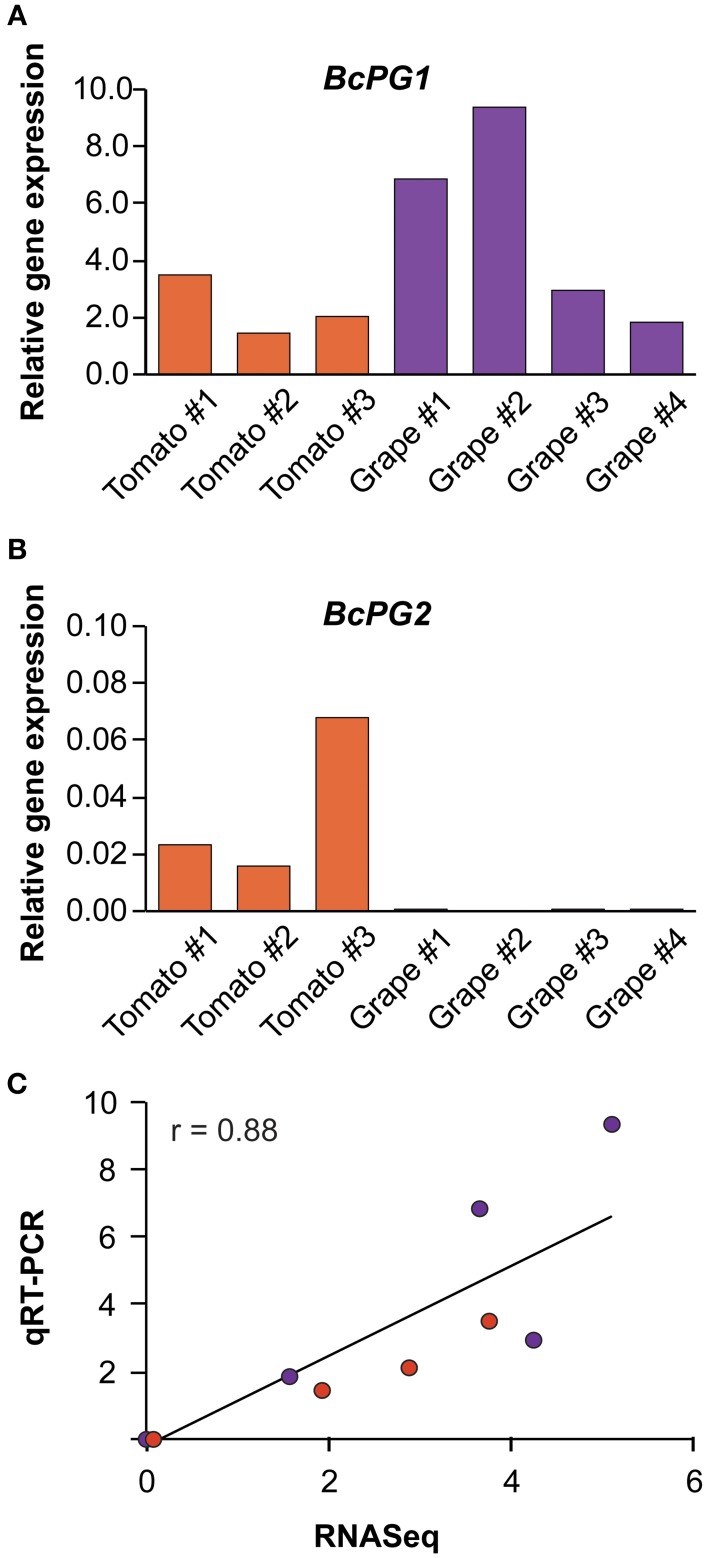
**qRT-PCR validation of the RNAseq expression results for *BcPG1* and BcPG2 genes in ripe tomato fruit and grape berries**. Relative expression levels of *BcPG1* **(A)** and *BcPG2* **(B)** measured in the biological replications of *Botrytis*-infected tomato fruit and botrytized-grape berries. **(C)** The scatterplot depicts the correlation between the qRT-PCR relative expression of *BcPG1* and *BcPG2* genes and their corresponding raw reads normalized against the same reference gene (*BcRPL5*) used in the qRT-PCR analyses. The data points indicate that the *BcPG2* expression in grape berries were close to zero (average of 4.49e−5) and in tomato fruit the expression was 3.57e−2 on average; thus they overlapped in the graph. The Pearson correlation coefficient (r) is presented (*P*-value < 0.001). A linear trend is shown.

*Botrytis* endo-PG (tribe 1) and PME (tribe 11) genes had the highest levels of expression (Figures 4A,C, Supplemental Table S2). However, not all the members of these tribes were expressed equally on the three plant hosts. Among the members of tribe 1, only the *BcPG1* gene was highly expressed in all hosts (Figure 4A). *BcPG3* had elevated expression in botrytized grape berries (1.28% of reads) but was expressed at lower levels in other infected host tissues (*P*-adjusted value <0.05); whereas the *BcPG6* gene was expressed more in infected lettuce leaves than on tomato fruit or grape berries (*P*-adjusted value <0.001), and *BcPG2* was not expressed during *Botrytis* infections of grape berries (*P*-adjusted value <0.001). The expression of the *BcPG1* and *BcPG2* genes were validated by quantitative reverse transcription PCR (qRT-PCR; Figure 5). A sequence alignment of the *BcPG2* gene of the B05.10 strain and the gene homolog of BcDW1 strain (primary inoculum used to induce noble rot in the grape berries; Blanco-Ulate et al., 2013a) indicated that they share 99.02% identity at the DNA level with no gaps. The mapping parameters used in this study were chosen to allow reads to map on a reference with a higher level of sequence diversity. To determine if sequence polymorphisms were responsible for the apparent lack of *BcPG2* expression, the RNAseq transcript reads from infected grapes were mapped to the predicted BcDW1 transcriptome and very similar mapping counts were obtained (*r* > 0.99; *P- value = 2.2e−16;* Supplemental Figure S2). The mapping coverage of *BcPG2* using BcDW1 as transcriptomics reference confirmed the absence of expression of this gene during *Botrytis* infections of ripe grape berries.

Although transcripts of all *BcPMEs* were abundant in all plant tissues, *Botrytis* selectively expressed specific PME genes in each host: *BcPME2* in lettuce leaves, *BcPME1* in tomato fruit and *BcPME3* in grape berries (Figure 4C, Supplemental Table S2). Hydrolases of the RG-I backbone (RGases; tribes 16 and 26) showed low levels of expression in all hosts, with the exception of *BC1G_05961* that was expressed at higher level in ripe fruit when compared to lettuce leaves (Figure 4A, *P*-adjusted value <0.001). On the other hand, *BC1G_01617* was the only exo-PG (tribe 10) with considerable levels of expression in all hosts; the highest level of expression of this gene was in lettuce leaves (*P*-adjusted value <0.001). Members of the PL1 and PL3 subfamilies, predicted to encode PLs and PELs, had low levels of expression (<0.3%) in all plant hosts. The exception was the *BcPL-like1* gene (tribe 8), which was more highly expressed in infected lettuce leaves than ripe tomato fruit (Figure 4B; *P*-adjusted value <0.05).

**Figure 6:**
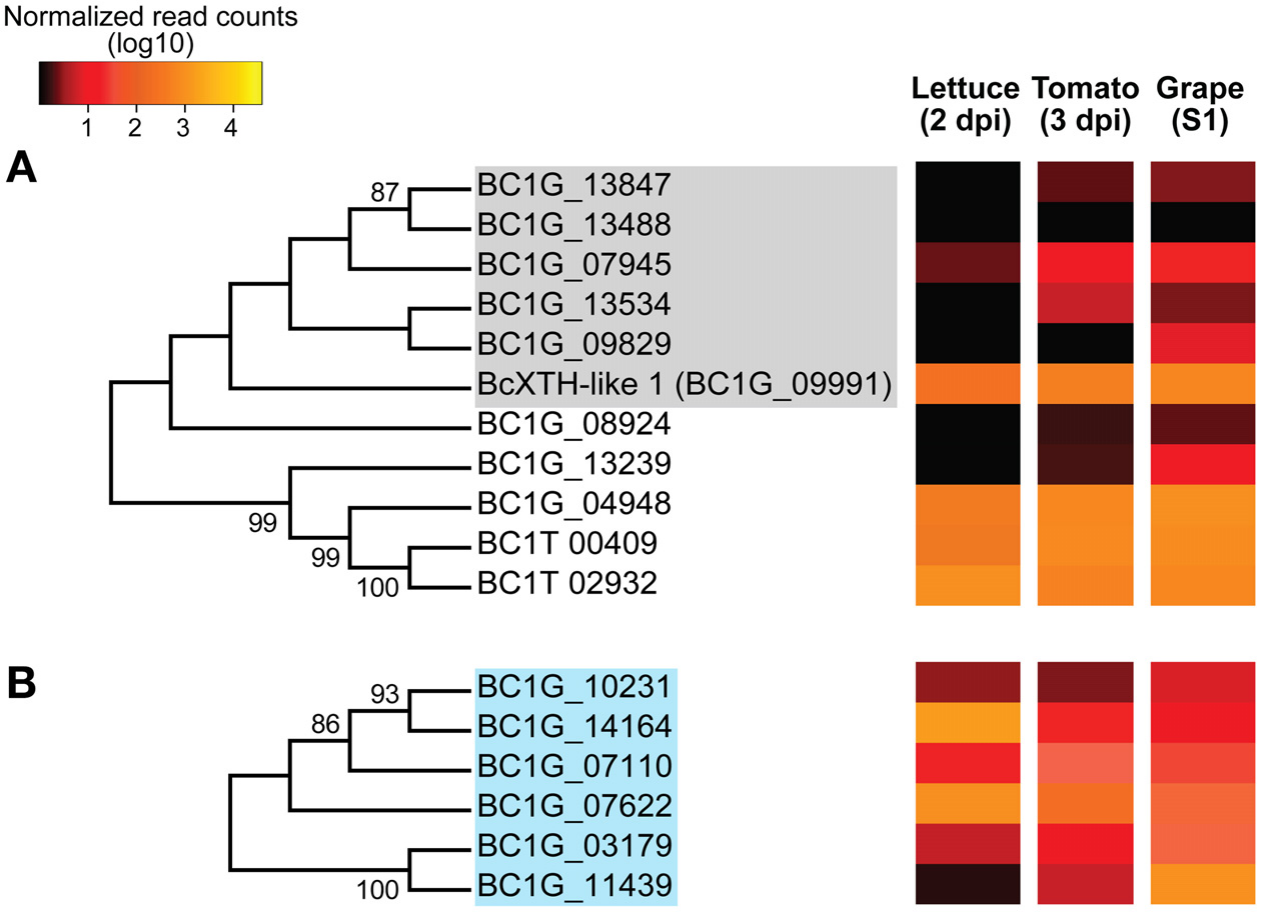
**Phylogenetic tree and expression levels of predicted xyloglucan (XyG) backbone-degrading enzymes**. Bootstrap consensus trees inferred from 1000 replicates using the Neighbor-Joining method for the *Botrytis* CAZymes with predicted signal peptides in the GH16 **(A)** and GH3 **(B)** subfamilies. The percentages (>50%) of replicate trees in which the associated taxa clustered together in the bootstrap test are shown next to the branches. Colored-boxes in the phylogenetic trees indicate protein tribes (> 2 members) determined by BLASTp alignments (*e*-value < 1e−6) and Tribe-MCL (Enright et al., 2002). Gray box in **(A)** correspond to tribe 5, while the light blue box in **(B)** refers to tribe 4 (Supplemental Table S2). The colors in the heat maps represent the number of DESeq-normalized transcript counts (log10) of the *Botrytis* genes in infected lettuce leaves, ripe tomato fruit and grape berries.

Figure 6 depicts the phylogenetic relationships among the CAZymes that might have roles in the breakdown of XyGs and cellulose. These include putative XTHs and glucanases (GH16 subfamily), and β-glucosidases (GH3 subfamily). The consensus phylogenetic tree of the GH16 subfamily separated the putative XTHs (tribe 5) from glucanases. Most candidate XTHs (tribe 5) showed low levels of expression in the three host tissues; e.g., the *BcXTH-like1* gene had an intermediate level of expression in ripe fruit tissues (>0.35% reads in both tomato and grape hosts). Three possible glucanases (*BC1G_00409*, *BC1G_04948*, and *BC1G_02932*) from diverse tribes have high expression in the three host tissues (Supplemental Table S2).

**Figure 7:**
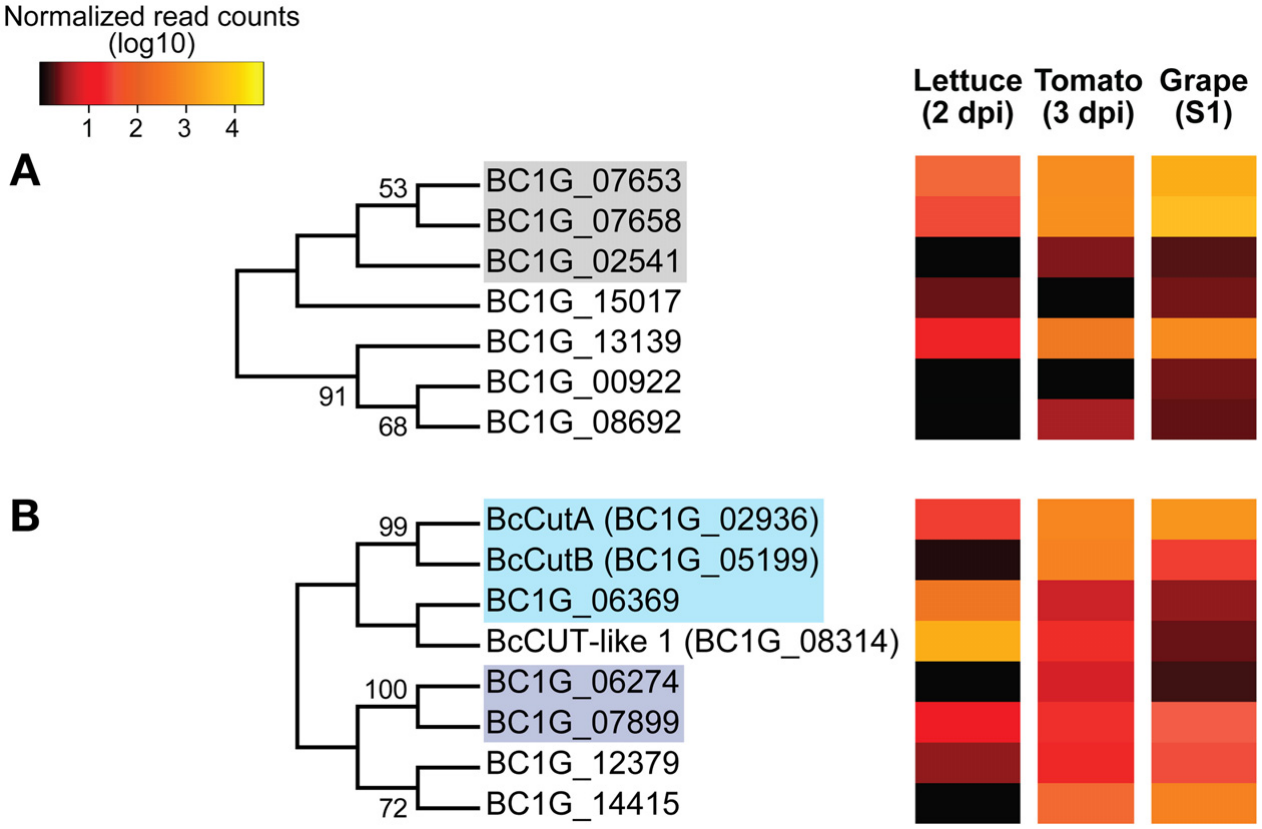
**Phylogenetic tree and expression levels of putative copper-dependent lytic polysaccharide monooxygenases (LPMOs) and cutinases**. Bootstrap consensus trees inferred from 1000 replicates using the Neighbor-Joining method for the *Botrytis* CAZymes with predicted signal peptides in the AA9 **(A)** and CE5 **(B)** subfamilies. The percentages (>50%) of replicate trees in which the associated taxa clustered together in the bootstrap test are shown next to the branches. Colored-boxes in the phylogenetic trees indicate protein tribes (>2 members) determined by BLASTp alignments (*e*-value < 1e−6) and Tribe-MCL (Enright et al., 2002). In **(A)**, gray box = tribe 13. Boxes in **(B)** light blue = tribe 15 and purple = tribe 31 (Supplemental Table S2). The colors in the heat maps represent the number of DESeq-normalized transcript counts (log10) of the *Botrytis* genes in infected lettuce leaves, ripe tomato fruit and grape berries.

The *Botrytis* proteins present in the AA9 subfamily include copper-dependent LPMOs proteins and other hypothetical proteins. Copper-dependent LPMOs are an auxiliary class of cell wall modifying proteins that may act on cellulose microfibrils. The phylogenetic analysis of the AA9 subfamily identified two potential clades, in one of which the LPMOs (tribe 13) grouped together. Two of these putative LPMOs (*BC1G_07653* and *BC1G_07658*) showed elevated expression in *Botrytis*-infected fruit, particularly in grape berries (Figure 7A; mentioned before).

Phylogenetic and gene expression analyses were done with proteins from the CE5 subfamily, some of which are involved in plant cuticle degradation (Figure 7B). There were two monophyletic groups in the consensus phylogenetic tree of the CE5 subfamily. Tribe 15, which included the cutinases, *BcCutA* (Van Kan et al., 1997) and *BcCutB* (Leroch et al., 2013), formed a separate group from the proteins of tribe 31, which included CAZymes without clear functional annotations. On the other hand, proteins from tribe 31 clustered together with putative acetylxylan esterases, which catalyze the deacetylation of xylan backbones, thus assisting, their subsequent degradation by hydrolytic enzymes. *BcCutA* gene was highly expressed (>0.45% of reads) by *Botrytis* in fruit tissues (*P*-adjusted value <0.001), while a putative cutinase gene (*BcCUT-like1*) was more highly expressed (2.03% of reads) during *Botrytis* infections of lettuce leaves (Figure 7B; *P*-adjusted value <0.001).

## Discussion

### *Botrytis* expresses a large and diverse set of enzymes to degrade plant cell walls

The presence and expression of specific CAZymes or gene families in plant pathogenic fungi have revealed the preferences of pathogens for particular host cell wall polysaccharides and infection strategies that are adapted to the pathogen lifestyle (King et al., 2011; Zhao et al., 2014). Our results indicate that *Botrytis*, a necrotroph, expressed most (>99%) of its 1155 CAZyme genes during infections of lettuce leaves, ripe tomato fruit and grape berries, indicating that expression of a large and diverse array of cell wall-targeting proteins is an important hallmark of the necrotrophic lifestyle.

*Botrytis*, as a generalist pathogen, infects a wide variety of host tissues with different cell wall compositions and architectures. Therefore, identifying enzymes that *Botrytis* produces on different hosts, can help to define parts of the host cell wall polysaccharide matrix that are important targets for *Botrytis* growth or metabolism. A common set of 229 CAZyme genes with secretion signals was expressed by *Botrytis* when infecting the three different hosts. Eighty-two of these proteins were also detected in proteomic studies of the *Botrytis* secretome (Shah et al., 2009a,b; Espino et al., 2010; Fernández-Acero et al., 2010; Li et al., 2012; Shah et al., 2012).

These common CAZymes probably constitute the core enzymatic machinery utilized by *Botrytis* as it penetrates and invades various plant tissues. Pectin-degrading enzymes (e.g., characterized endo-PGs, putative RGases and candidate exo-PGs) were the most abundant plant cell wall modifying proteins among the CAZymes expressed in all hosts. This result confirms that pectin degradation is a key process during *Botrytis* infections of plant tissues, including those with higher proportions of cellulose and hemicellulose polysaccharides, such as lettuce leaves (Nunan et al., 1998; Wagstaff et al., 2010; Lunn et al., 2013).

*Botrytis* adapts its infection strategy to the diverse conditions present in the host cell walls. The expression of 43 *Botrytis* genes encoding predicted secreted CAZymes was detected in some but not all plant hosts. The majority (83.72%) of these genes were commonly expressed in tomato and grape tissues, but not in lettuce leaves. Among these fruit-specific genes, those encoding putative xyloglucan (XyG) transglucosylase/hydrolases and glucanases were the most abundant. A candidate exo-PG (*BC1G_13137*) gene that is only detected when in *Botrytis*-infected fruit had been detected when this pathogen was grown in minimal medium supplemented with pectins as the sole carbon source, which suggests that this enzyme is important in the degradation of host cell walls that are rich in pectins (Shah et al., 2009a). The molecules or signals lead to the expression of host-specific enzymes are not known, but some could result from the degradation of host cell walls by core CAZymes (e.g., pectin derived oligosaccharides; Körner et al., 1998; An et al., 2005).

*Botrytis* may promote susceptibility in the host tissues by inducing or suppressing the expression of plant cell wall degrading enzymes (AbuQamar et al., 2006; Flors et al., 2007; Cantu et al., 2008b, 2009a). Fungal and plant enzymes may cooperate to effectively digest relatively complex polysaccharides. In addition, the activity of plant enzymes can result in the disassembly of the host cell walls beyond the site of fungal infection, which would facilitate the subsequent growth of *Botrytis* into the host tissues.

### Pectin modifying enzymes

*Botrytis* produces a large collection of enzymes to degrade the backbones and side-branches of pectin polysaccharides. Breakdown of pectins during infections increases the plant cell wall's porosity, and may facilitate the degradation of other wall polysaccharides and the growth of *Botrytis*. Enzymes that target pectin backbones include PGs and RGases (GH28 subfamily), and PLs/PELs (PL1 and PL3 subfamilies). PMEs (CE8 subfamily) and RG esterases (CE12 subfamily) might cooperate in the effective degradation of pectin backbones (Van Kan, 2006; Zhang and van Kan, 2013a).

PGs hydrolyze the backbones of HGs. The *Botrytis* genome encodes 11 potentially secreted PGs, of which at least five are likely exo-PGs (i.e., predicted to remove sugar monomers from the non-reducing ends of HG or oligomeric products generated by endo-PG action on HG) and six are endo-PGs (i.e., hydrolyze the HG polymer at internal sites). When infecting lettuce leaves and ripe fruit, *Botrytis* expressed four exo-PG and five endo-PG genes (Table 2). *BcPG2* was not detected in botrytized grape berries. The expression of *BcPG2* was up-regulated in the presence of pectate as carbon source in a host-free transcriptomics analysis (Zhang et al., 2013). The BcPG2 protein was detected at high levels when *Botrytis* was grown in media enriched with partially esterified pectin, glucose and extracts of tomato fruit, while only small quantities of BcPG2 protein were observed in *Botrytis* cultures supplemented with highly esterified pectin substrates or kiwi fruit extracts (Shah et al., 2009a; Espino et al., 2010). Taken together, these results indicate that the expression of *BcPG2* is differentially regulated depending on the conditions present in the plant host's cell wall matrixes (e.g., esterification status of the pectin polysaccharides; presence of inhibitors or activators of *BcPG2* gene expression, availability of particular cell wall substrates, efficiency of other BcPGs in a particular tissue).

In agreement with previous reports, *BcPG1* was the most highly expressed CAZyme gene in lettuce leaves, ripe tomato fruit and grape fruit (Reignault et al., 2000; Wubben et al., 2000; Ten Have et al., 2001). Although BcPG1 is not indispensable for virulence, the Δ*bcpg1* knockout mutant is significantly less virulent in diverse hosts (Ten Have et al., 1998; Zhang and van Kan, 2013b). The expression data in this study support the conclusion that PGs, especially BcPG1, have major roles for *Botrytis* infections in a broad-range of plant tissues (Ten Have et al., 1998, 2001; Powell et al., 2000; Kars et al., 2005a; Rowe and Kliebenstein, 2007; Zhang et al., 2014).

Plants produce PG inhibiting proteins, PGIPs, to reduce the extensive pectin degradation caused by fungal, bacterial or insect attack (De Lorenzo et al., 2001). PGIPs inhibit most of the *Botrytis*'s PGs (Sharrock and Labavitch, 1994; Joubert et al., 2007). Over-expression of PGIPs has been proven to increase the abundance of this inhibitor in the cell wall matrix and to reduce *Botrytis* growth on vegetative tissues and ripe tomato fruit (Powell et al., 2000; Ferrari et al., 2003, 2006).

In addition to the production of its own endo-PGs, *Botrytis* can trigger the expression of plant genes encoding endo-PGs. Because plant endo-PGs are not inhibited by PGIPs (Cervone et al., 1990), they may assist in the breakdown of host cell wall pectins even when inhibitors of fungal enzymes are present. The tomato endo-PG, *SlPG2A*, is precociously up-regulated by *Botrytis* infections of unripe tomato fruit (Cantu et al., 2009a; Shah et al., 2012). The *SlPG2A* is considered a key cell wall degrading enzyme during tomato fruit ripening and softening (Bennett and Labavitch, 2008; Cantu et al., 2008b), and thus, its premature induction may benefit *Botrytis* infections.

PLs and PELs degrade HGs by a β-elimination rather than by hydrolysis. PLs generally act on heavily methylesterified HG backbones, and PELs are more efficient on lightly methylesterified HGs. Four PLs and four genes encoding PELs were annotated in the *Botrytis* genome, and the expression of all was detected in the three hosts (Table 2). However, the expression of these genes was lower than the expression of the *BcPG* genes, suggesting that *Botrytis* PLs/PELs assist PGs and are not the primary enzymes attacking HGs.

The extent of methylation and acetylation of the HG backbones can impact the activity of *Botrytis* endo-PGs and PELs (Kars et al., 2005a). PMEs catalyze the specific demethylesterification of HGs. Three putative secreted PMEs are present in the *Botrytis* genome (*BcPME1-3*; Kars et al., 2005b). Although these three PMEs were expressed in *Botrytis*-infected lettuce leaves, tomato fruit, and grape berries, *Botrytis* PME activity seems not to be essential for virulence on certain hosts. Knockout mutations in *BcPME1* and *BcPME2* did not affect *Botrytis*'s virulence on leaves of tomato and grapevine and on pear fruit (Kars et al., 2005b). However, BcPME1 was necessary for successful infections of apple fruit (Valette-Collet et al., 2003). It is possible that *Botrytis* relies on plant PMEs for demethylesterification of the HG backbones in certain host tissues (Raiola et al., 2011), or that the activity of BcPME3 can compensate for the absence of BcPME1 and BcPME2 (Kars et al., 2005b).

Plant PMEs may act as susceptibility factors by cooperating with *Botrytis* PMEs for the demethylesterification of HG backbones (Lionetti et al., 2012). For example, infection of Arabidopsis leaves by *Botrytis* alters the expression of host PME genes (AbuQamar et al., 2006), and the enhanced gene expression and activity of AtPME3 increases susceptibility to *Botrytis* (Raiola et al., 2011). In an effort to counteract the increased PME activity that results from encounters with pathogens, plants produce PME inhibitors (PMEIs; An et al., 2008; Volpi et al., 2011; Lionetti et al., 2012, 2014). In vegetative tissues, the over-expression of plant PMEIs had been effective for limiting *Botrytis* infections Lionetti et al., 2007, 2012.

The *Botrytis* genome includes six possible secreted RGases, two α-L-rhamnosidases and an RG acetylesterase (Table 2), which could cleave or modify RG-I backbones (Schols et al., 1994; Mutter et al., 1998; Mølgaard et al., 2000). Although expression of most of these genes was detected in the *Botrytis*-infected tissues, the low level of their expression may reflect the paucity of RG-I compared to HG pectins. RG-I is a major part of the hairy region of pectins in plant cell walls, but they are not as abundant as pectins with HG-backbones (Voragen et al., 2009).

*Botrytis* expressed diverse enzymes that are predicted to degrade pectin side-branches. Among these genes were four α-arabinofuranosidases, three β-galactosidases, and two α-L-1,5-arabinanases (including *BcAra1*; Table 2). BcAra1 has been shown to degrade 1,5-arabinan *in vitro*. On Arabidopsis leaves but not tobacco or tomato leaves, the Δ*bcara1* knockout mutant has reduced virulence (Nafisi et al., 2014).

### Hemicellulose modifying enzymes

A variety of hemicellulose-modifying enzymes is encoded in the *Botrytis*'s genome. XyG backbones are hydrolyzed by endo-acting β-1,4-glucanases or β-glucosidases, which also act on cellulose (Gilbert, 2010). All of the predicted *Botrytis* β-glucosidase genes were expressed in infected lettuce leaves, tomato fruit and grape berries; however, expression levels of some were higher on lettuce leaves (Supplemental Table S2). The β-glucosidases were among the most numerous glycosyl hydrolases in fungal genomes (Zhao et al., 2014). XTHs can act on XyG backbones. They have two possible catalytic activities: (1) XyG endo-transglucosylase (XET) activity, which results in the non-hydrolytic cleavage and ligation of XyG polymers, and (2) XyG endo-hydrolase (XEH) activity that leads to the irreversible shortening of the XyG backbone (Eklöf and Brumer, 2010). The *Botrytis* genome has six candidate XTHs (GH16 subfamily), and two of this genes (*BcXTH-like1* and *BC1G_07945*) were commonly expressed at low levels in all of the host tissues studied.

Xylans and mannans are present in the primary and secondary walls of many of *Botrytis's* hosts, but they are less abundant than XyGs. Digestion of these hemicelluloses may be important for *Botrytis*'s energy acquisition and tissue exploration efforts. Expression of four β-xylanases (including *BcXyn11A*; Table 2) and two β-xylosidases (*BC1G_02487* and *BC1G_10797*), which target xylan backbones, is detected when *Botrytis* infects lettuce leaves, ripe tomato fruit and post-véraison grape berries. Deletion of the *BcXyn11A* gene delayed disease symptoms and reduced the lesion size on tomato leaves and table grape berries (Brito et al., 2006). However, the contribution of BcXyn11A to overall virulence does not depend on its xylanase activity; rather, it is related to the necrosis in the host caused by the xylanase protein itself (Noda et al., 2010).

Some of the side-branches along the XyG and xylan backbones contribute to the overall strength of the hemicellulose-cellulose microfibril network (Pauly et al., 2013). Therefore, removal of these groups might affect the hemicellulose cross-linking properties and, at least locally, disrupt the wall's hemicellulose-cellulose network. In all hosts analyzed, *Botrytis* expressed two α-xylosidases (*BC1G_12859* and *BC1G_11115*) and one α-L-fucosidase (*BC1G_08975*). These enzymes could digest XyG side groups, exposing the hemicellulose's glucan backbone to further digestion. Additional enzymes that remove side-branches in hemicelluloses may be the same as or functionally equivalent to CAZymes that trim the side groups of pectins; e.g., α-arabinofuranosidases (previously described).

### Cellulose modifying enzymes

*Botrytis* expresses genes encoding predicted cellulose-degrading enzymes; these include nine endo-β-1,4-glucanases (including *BcCel5A*), five cellobiohydrolases, and the previously discussed β-glucosidases. Espino et al. (2005) demonstrated that a mutant with a deletion in *BcCel5A* (*BC1G_00642*), an endo-β - 1,4-glucanase encoding gene, can infect tomato leaves and gerbera petals. Because the expression of *BcCel5A* appears to be relevant during spore germination and penetration of waxy surfaces (Leroch et al., 2013), evaluating the virulence of Δ*bccel5a* mutants in these hosts may provide information about the importance of this enzyme during infections.

As consequence of *Botrytis* infection, expression of plant endo-β-1,4-glucanases is reduced (Flors et al., 2007; Finiti et al., 2013). Transgenic suppression of endo-β-1,4-glucanases limited *Botrytis* growth and promoted the activation of defense responses in tomato fruit and Arabidopsis and tomato leaf tissues. These responses included enhanced callose deposition and expression of defense genes, e.g., *PR1* and *LoxD* (Flors et al., 2007; Finiti et al., 2013).

Copper-dependent lytic polysaccharide monooxygenases (LPMOs) cooperate with canonical cellulose-degrading enzymes and other electron transfer proteins to accelerate the degradation of cellulose microfibrils (Hemsworth et al., 2013). A number of LPMOs have been identified mainly in fungal genomes, especially in wood decay-causing fungi (Levasseur et al., 2013). *Botrytis* expressed two putative LPMOs (*BC1G_07653* and *BC1G_08692*) in lettuce leaves and fruit hosts. However, a larger number of LPMOs was expressed during infections of ripe fruit (especially grape tissues) and their expression was higher than in the other plant host tissues (Supplemental Table S2).

### Cutinases

In the absence of cracks or wounds in the plant surface, the initial interactions between a host and *Botrytis* occur at the plant cuticle. In those situations, *Botrytis* secretes an assortment of cutinases and lipases to breach the cuticle and penetrate the host (Van Kan et al., 1997; Reis et al., 2005; Van Kan, 2006; Leroch et al., 2013). Cutinases cleave ester bonds between cutin monomers (Pio and Macedo, 2009).

When infecting the three hosts analyzed, *Botrytis* expressed *BcCutA* and three other putative cutinases (*BcCutB, BcCUT-like1* and *BC1G_06369*). The expression levels of these enzymes depended on the host tissues; for example, *BcCutA* was highly expressed in fruit, but not in lettuce leaves. The expression of *BcCutA* was up-regulated during early germination of *Botrytis* spores on apple wax; this may indicate that *BcCutA* may be preferentially expressed at the surfaces of fleshy fruit, where wax accumulation is common (Leroch et al., 2013). Some *Botrytis* cutinases showed high homology to acetylxylan esterases, which are associated with the degradation of xylans. The *Botrytis* genome encodes two putative acetylxylan esterases (*BC1G_12379* and *BC1G_07899*), and one was expressed in all infected plant tissues analyzed. Results in Skamnioti et al. (2008) suggested that the functional diversification between cutinases and acetylxylan esterases may have occurred before the speciation of *Botrytis cinerea* and two other ascomycete pathogens, *Fusarium graminearum* and *Neurospora crassa*.

## Concluding remarks

The diversity of CAZyme-encoding genes in the *Botrytis* genome and their extensive expression when this necrotroph interacts with its hosts suggests that the pathogen's ability to degrade a wide-range of cell wall polysaccharides is tightly associated with its success in infecting a broad range of plant hosts. While several proteins were previously identified by proteomic analyses, this study expands the catalog of the complex array of enzymes that *Botrytis* may secrete to digest host tissues. Pectins, particularly pectin backbones, appear to be the main target of degradation by *Botrytis* in leaf and fruit tissues. However, *Botrytis* also expresses particular CAZyme genes only when infecting certain hosts. What promotes this host-specific expression of CAZyme genes is not known. Information about the structural details of the associations between the constituents of diverse host plant cell walls is needed to fully understand how *Botrytis* benefits from the digestion of plant cell wall polysaccharides during successful infections. In addition, understanding plant responses to *Botrytis* infections, which may include altered expression of endogenous CAZyme genes (Flors et al., 2007; Cantu et al., 2008b, 2009a) or inhibitors of cell wall degrading enzymes (De Lorenzo et al., 1994; Powell et al., 2000; De Lorenzo and Ferrari, 2002; Lionetti et al., 2007, 2014) and plant cell wall fortifications (Van Baarlen et al., 2007; Finiti et al., 2013), may shed some light on the co-evolution of plant and pathogen strategies and their impact on resistance or susceptibility to fungal infections.

Measurements of enzymatic activity *in vitro* as well as *in planta* on cell wall polysaccharides may confirm the predicted enzymatic activities of some of the genes described in this study. This information may refine our understanding of important virulence functions needed for successful *Botrytis* infections. Another strategy to demonstrate that cell wall modifications have occurred during *Botrytis* infection would be to identify the accumulation of characteristic breakdown products; e.g., pectin derived oligosaccharides that result from the activity of PLs and PGs (Melotto et al., 1994; Körner et al., 1998; An et al., 2005). Validation of the role and function of the *Botrytis* cell wall modifying enzymes may also be achieved by targeted mutagenesis, with the caveat that it is expected that most of these enzymes have paralogs with redundant activities and/or their functions may depend on other proteins, including some produced by the plant host.

### Conflict of interest statement

The authors declare that the research was conducted in the absence of any commercial or financial relationships that could be construed as a potential conflict of interest.
