# Supplementary material for: Genome-wide transcriptional profiling of Botrytis cinerea genes targeting plant cell walls during infections of different hosts
Source: Front Plant Sci. 2014 Sep 3;5:435. doi: 10.3389/fpls.2014.00435 (PMC4153048; doi:10.3389/fpls.2014.00435)
Supplement: Supplementary file 1 [file Presentation1.ZIP › Supp Mat Figure 2.PDF]

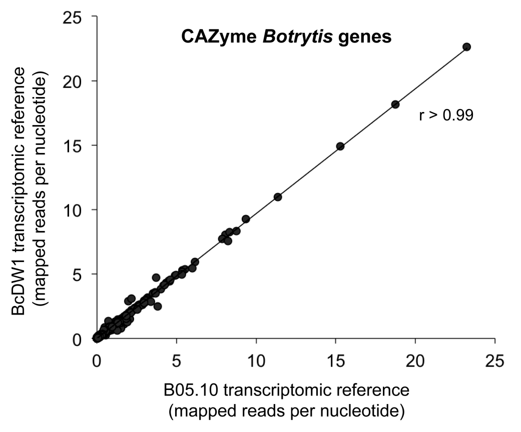
CAZyme *Botrytis* genes
r > 0.99
BcDW1 transcriptomic reference
(mapped reads per nucleotide)
B05.10 transcriptomic reference
(mapped reads per nucleotide)
0
5
10
15
20
25
