# Supplementary material for: Genome-wide transcriptional profiling of Botrytis cinerea genes targeting plant cell walls during infections of different hosts
Source: Front Plant Sci. 2014 Sep 3;5:435. doi: 10.3389/fpls.2014.00435 (PMC4153048; doi:10.3389/fpls.2014.00435)
Supplement: Supplementary file 1 [file Presentation1.ZIP › Supp Mat figures & tables captions.pdf]

Supplemental Online Material - captions

Figure S1. Correlation between *Botrytis* biomass and RNAseq reads uniquely mapped to *Botrytis*. Scatterplot shows the correlation between the amount of *Botrytis* biomass (µg/FW of fruit tissue) and number of *Botrytis* transcript reads present in infected tomato and botrytized grape berries. Linear trends and Pearson's correlation coefficients (r) are shown.

Figure S2. Mapping of CAZyme transcript reads from botrytized grape berries to the *Botrytis* B05.10 and BcDW1 transcriptomes. Scatterplot shows the correlation between the CAZyme *Botrytis* transcripts from infected grape berries mapped to the predicted transcriptomes of the strain B05.10 (Amselem et al. 2011) and BcDW1 (Blanco-Ulate et al. 2013a). In both cases combined transcriptomes of *Botrytis*, B05.10 or BcDW1, and grape (v. 12X) were used as references to increase the specificity to fungal genes. Only BcDW1 genes that are predicted homologs of B05.10 genes by BLASTn (> 98% of identity and > 95% subject coverage) were used in this analysis. A linear trend is depicted and the Pearson's correlation coefficient (r) is shown.

Table S1. Transcript counts for the 1,155 CAZyme genes present in the *Botrytis cinerea* (strain B05.10) genome during infections of three plant hosts. Presented are gene accessions (http://www.broadinstitute.org/annotation/genome/botrytis_cinerea), CAZyme subfamilies, protein tribes, the functional annotations and the transcript counts per infected host tissue (i.e., lettuce leaves, tomato fruit and grape berries) for all *Botrytis* CAZymes. *Botrytis* transcript counts from infected lettuce leaves were obtained from De Cremer et al. (2013).

Table S2. Expression levels of *Botrytis cinerea* (strain B05.10) genes encoding putative secreted CAZymes in infected lettuce leaves, ripe tomato fruit and grape berries. The table includes the 275 CAZyme *Botrytis* genes with secretion signals, their accessions (http://www.broadinstitute.org/annotation/genome/botrytis_cinerea), CAZyme subfamilies, protein tribes, functional annotations, DESeq-normalized transcript counts and their relative read percentages from the total normalized reads of the 275 *Botrytis* genes in a given plant host. The table specifies the expression fold changes (log2) of the CAZyme genes and their correspondent *P* adjusted values when comparing the three infected plant tissues (i.e., tomato fruit/lettuce leaves, grape berries/lettuce leaves and grape berries/tomato fruit) using DESeq. In addition, the table indicates if the proteins encoded by these CAZyme genes have been detected in any of the published proteomic studies of the *Botrytis* secretomes (Shah et al. 2009a and 2009b; Espino et al. 2010; Fernández-Acero et al. 2010; Shah et al. 2012; Li et al. 2012).
