# Supplementary figures and images for: Genome-wide transcriptional profiling of Botrytis cinerea genes targeting plant cell walls during infections of different hosts
Source: Front Plant Sci. 2014 Sep 3;5:435. doi: 10.3389/fpls.2014.00435 (PMC4153048; doi:10.3389/fpls.2014.00435)

## Tomato

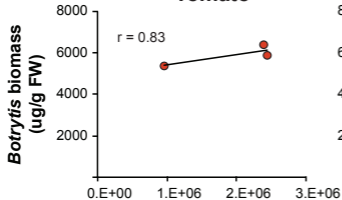

## Grape

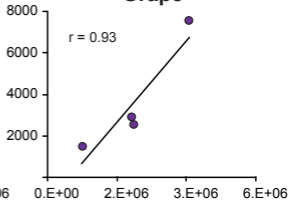

Mapped reads to *Botrytis* reference

Supplement: Supplementary file 1 [file Presentation1.ZIP › Supp Mat Figure 1.PDF]
